# Vaginal Celiotomy1Read at the twenty-eighth annual meeting of the Medical Association of Central New York, at Syracuse, October 15, 1895.

**Published:** 1896-04

**Authors:** William B. Jones

**Affiliations:** Rochester, N. Y.; 215 Lake Avenue


### VAGINAL CELIOTOMY.[1]

By WILLIAM B. JONES, M. D., Rochester, N. Y.

IN VAGINAL celiotomy we in great measure avoid sepsis, shock and hemorrhage. As to sepsis, let me remind you that a part of the peritoneum becoming infected does not cause general septic peritonitis if the discharges from it are not forced over the rest, as seen in operation by two stages of colotomy or for artificial anus; or better, in appendicitis if abscess bursts after adhesions are formed, or still more nearly like the conditions in this opera-

1. Read at the twenty-eighth annual meeting of the Medical Association of Central New York, at Syracuse, October 15, 1895.

tion, if it bursts without such adhesions; after that general peritonitis will be prevented, always, I believe, by leaving open a free incision and draining continually with gauze. Notice that such drainage lacks several advantages of the vaginal form.

In the latter, the abdomen is opened in its most dependent part, the intestines frequently are not seen, they are never disturbed and if any become infected they are not pushed away among other coils. Below them there is the hollow of the sacrum and coccyx, an ideal trap where all oozing collects and is emptied by the vaginal tent on to the external dressing, being disinfected as it goes. The amount of oozing is a revelation to everyone. To see it, is to marvel at the amount the peritoneum has to absorb when it is not drained. It is the perfection of culture media for septic germs, and where it lingers is a forcing bed for their development. The advantage of removing it is obvious.

Now let us consider the course of an infection of a vaginal celiotomy wound; inflammation begins, exudation of serum and lymph occurs, serum drains into Douglas's pouch, whence it is immediately disinfected and removed, the lymph forms adhesions everywhere, roofing over the septic area and building a barrier that stops the process. It is interesting to watch the temperature during this time. On the second day, in severe cases, it rises rapidly during the few hours required for checking the progress of inflammation, then it rapidly falls to nearly normal and remains there. Easy cases without that inflammation do not have that rise.

This is not the place to discuss indications for hysterectomy, but all will advise that all tissue irreparably diseased should be removed, uterine or other, and what is removed in no way affects the principle of operation. A hysterectomy makes it easier.

Freedom from shock is due to the fact that you do not drag out the intestines and expose them to air and changes of temperature, you do not make a roadway through them to be traversed many times by hands and instruments with whatever they have to carry, you do not handle the intestines at all in most cases, scarcely at all in any, and then through some distance of canal that allows no change of temperature. The patient's abdomen is kept warm and covered instead of being exposed, wet and cold.

The uterine and ovarian arteries should be found and tied as early as practicable in cases of large solid tumor and on the affected side in ectopic gestation. Otherwise no ligatures are necessary, except on the pedicle. Before they can be reached, strong traction on the tumor is effective in preventing bleeding, by compression. After operation, hemorrhage cannot occur without the nurse knowing it from the start, and this is a strong point in the operation.

Every drop of blood and other discharge, too, starts for the outer world directly it escapes from the vessels or tissues and the dressings surely indicate what is going on. Furthermore, in case of primary hemorrhage that had been overlooked, or of secondary hemorrhage, there is no new operation to perform. Clean your hands and pass a clamp to the bleeding point, close it and the bleeding stops. The clamp stays forty-eight hours and the patient recovers. Hemorrhage, after a closed wound, is not discovered until the patient is *in extremis* ; to stop it requires another operation, especially formidable at that time, and it is true that few recover.

I have shown that patients should not die from sepsis, shock or hemorrhage. If they do not die from these, they do not die, and so sure of this am I that, when it is my own patient, I frequently say to her or her friends beforehand that if it is not a septic case to begin with and she should die it would require an explanation. It could not be from an operation itself, properly performed. I allow a little leeway in pus cases, because I am not sure that in certain cachectic and diathetic conditions we can invariably prevent absorption through lymphatics, even in a wide-open wound. I think it may possibly occur, but do not know that it can.

I would not commit myself to that statement in all large tumors either, because in extremely difficult ones shock or hemorrhage might be possible, although, if they are operable from below, never so probable as by laparatomy from above. These two exceptions are the only ones.

Intestinal obstruction is caused by adhesions. Old ones may be broken up and new ones formed in new places. A drainage-tube always causes adhesions in its vicinity ; a pedicle ligated and dropped does, temporarily at least, and often permanently if the ligature irritates, with or without being infected. In the case of infected ligature a sinus follows, further increasing the amount of adhesions and, remaining open, causes suffering and annoyance for weeks, or months, or forever. Operations to remove such ligatures are occasionally required, and sometimes fail. Fecal and vesical fistulæ sometimes complicate such a sinus, and when the whole trouble is closed up, the adhesions it has caused are extensive and there is more or less pain and disability while the patient lives. We avoid all these causes and results of adhesions, except in small degree, the freeing of old adhesions and allowing the bowel to form new attachments. As a rule, before operation the intestines are already in a better position than we can put them by any change. If we do not have to break them up to go through to operate on what is below, we can leave them as they are. There was not obstruction before, there will not be afterward, and with the removal of the focus of irritation, part of the old adhesions will be absorbed.

One case has been reported, making an unnecessary exception. The bowel became adherent to the wound and obstructed. The operator states, as any one can understand, that had he made a digital examination and released it there would have been no trouble. It illustrates a point I make later in my paper, that whatever occurs during convalescence is easily accessible all the time, and all operators look back to the times when something went wrong and they would have given anything to know just how the site of operation was.

Less anesthetic is used. Complete relaxation is not required, but only absolute insensibility. I am sure we do not sufficiently realise how much of prostration, nausea, faintness and suppression of urine are due to ether or chloroform intoxication. We agree that anesthesia is attended with slight danger of sudden death, and recently we are coming to know that other bad effects are present in nearly every case and increase directly with the dose given.

Stitch abscesses are out of the question, of course, and as we have no mural abscesses or drainage through the abdominal wall and as there is no abdominal incision, hernia also is an impossibility. The frequency of hernia in laparatomy wounds can never be known, but it occurs with every operator, by every method of closing the wall, even by permanent sutures, and they frequently irritate. Abdominal scar itself is not so great a detriment, yet any woman or man will be pleased to do without it. Pain, vomiting, thirst and prostration during the first hours are infinitely less than after laparatomy. The woman is nearly always comfortable as soon as she is really conscious and on the second day she is contentedly resting, taking abundant nourishment and growing strong. At the end of the third or fourth day she sits up in bed, next day she is out of it, and goes home some time between the fifth and fourteenth. Two weeks is the longest period of hospital care; five days the shortest. Please compare that with other celiotomies.

The complexion and facial expression, right from the first, are almost normal. There is none of the pale skin and anxious look or lines of pain. None of the faintness, nausea and utter prostration, so near impending dissolution, in the worst cases. The temperature, on the whole, ranges lower too. There is no troublesome tympanites.

All the pelvis can be easily palpated, part of it more readily than from above, and nearly all of it can be seen. Tubes and ovaries changed by disease can be removed or conservative work upon them can be done. Fluid tumors of all sizes, including abscesses, are operable. Solid tumors reaching to any height below the umbilicus can be taken out. Extrauterine pregnancy is accessible, and masses of adhesions already infected afford the most advantageous condition for preferring the vaginal route. It shows all its points of superiority in a case of universal adhesions with multiple abscesses and sinuses honeycombing in all directions, some of them communicating with intestine or bladder. Ordinarily such patients die from chronic sepsis and exhaustion or from acute sepsis if operated upon. But open from below, remove as much as possible of the disease, leave the perfect drainage, never attainable except through the vagina. All will heal in *a few days* and the woman goes home in two weeks cured, except that the intestinal or vesical fistula when present nearly always heal in two to six weeks. I am speaking of cases practically inoperable from above. They get well. "The extremity of the laparatomist becomes the opportunity of the vaginal celiotomist." Hysterectomy can be done, of course, and is safer by vagina. Small tumors can be taken from any part of the uterus within, or upon any portion of it and the organ left sound and whole, instead of their being temporised with until they make hysterectomy necessary. Thomas's spoon saw is almost abandoned for submucous growths, because of the danger of its going clear through and causing fatal peritonitis or laceration of intestine. With two fingers applied through the vaginal vault over the site of operation, it can be used with impunity even if the growth is so deeply seated as to require resection of the whole thickness of the uterine wall.

Subserous growths can be taken from anywhere on the uterine surface. Ovarian cysts of large size can be emptied and extracted the same as through any other opening. Small ones are better for this method. Very firm adhesions high up may make another way preferable. If universal adhesions make it impracticable to remove one by any method, it can be opened per vaginam, emptied, and packed and drained with gauze, to be obliterated by granulation more safely than by any other treatment. Large hard tumors of pelvic origin if liable to bleed would be more rapidly and safely removed by laparatomy, if all vessels and tissues to be reached from below were first ligated and divided that way. Not very long ago I lost such a patient from hemorrhage and shock who would be alive today if we had been doing that at that time.

Anteflexion of the uterus has no satisfactory treatment for most of the cases that need it. It is mainly due to attachments of the bladder so highly anteriorly that the fundus of the uterus is drawn forward and downward. On the 26th of last June I operated for this condition upon a patient who had dysmenorrhea since puberty. After thorough dilatation, curettage and tamponade the organ resumed its malposition immediately. Thereupon I opened the anterior vaginal vault, severed all anterior attachments and it straightened out at once like a steel spring. I have examined the patient frequently since and she is anatomically and symptomatically cured. Theoretically the method is correct and it has the evidence of one successful case toward proof, practically. It is original with me, though others probably have thought of it before I did. In the New York *Medical Record* for July 20, 1895, Dr. William R. Pryor describes an operation on similar principles for retro-displacements. Vaginal tamponade is an essential part of after treatment and results have been extremely satisfactory. I can give testimony from my own experience in its favor.

All the operations can be done for acute sepsis, puerperal or otherwise, in uterus, tubes, ovaries, pelvic peritoneum and cellular tissue, enabling us to stop this process in the beginning and avert prolonged suffering, with destruction of important organs, or save the life of the woman, avoiding the necessity of operation later for incurable disease. The importance of this is beyond comprehension. Ninety per cent. of mutilating disease of female generative organs begins as acute sepsis, begins as inflammation, localised, but very soon to destroy a most important organ or more than one. A phlegmon anywhere else endangering a vital part would be opened early, drained of serum and poisoned blood; inflammation would stop where it was and neighboring tissues be saved from destruction. The same can be done by vaginal celiotomy, as soon as pus is formed, even before that. If it be done, a large proportion of the present gynecic surgery will not be needed, a great deal of this disease will be averted. To a far greater extent than now will it be the healing art instead of the last resort of removal of what we cannot cure.

In many cases the procedure is slow and in all it lacks every dramatic element of a brilliant, rapid operation, but that can be no criticism when we consider its superb results and there is almost never a tragedy upon which to drop the curtain.

Contrary to a general impression it is not working in the dark. Every step of an easy case may be done under the senses of vision and touch. Difficult ones must be done in part by tactile sense alone, but that is equally true of other methods; and afterward all processes of repair with possible accidents remain accessible all the time, so that during the most important period all is in the light when otherwise it would not be.

Injury to intestines, ureters and bladder are less liable to occur, because it is easier to push them out of the way and keep them there and because it is not necessary to go through an adherent mass of them to arrive at the place of the operation proper.

"It is difficult," yes, and to this objection I willingly assent, but shall we reject a superior measure because it sometimes requires greater skill? There have never been such results as this operation is demonstrating. Many have been restored to health who would have died with our best skill. Jacobs, of Belgium, has had 413 cases, including every condition that can occur within a woman's pelvis, excluding those not serious enough to require total extirpation of uterus, tubes and ovaries, and there were only twelve deaths. Pozzi has had 144 consecutive nonmalignant cases all recovered.

If there is any way by which better work can be done, I want to learn it and give up this. There never has been any approach to such results.

I wish to be understood that it is not suitable for all conditions, that some should never be attempted from below, and circumstances will occasionally make it necessary to abandon work already begun and finish it from above, and that no one should do it at all who is not already familiar with ordinary laparatomy and prepared to change from one to the other.

There are four cardinal reasons for vaginal celiotomy. You avoid sepsis, shock, hemorrhage and intestinal obstruction. They are not the only ones I urge, but discard all the rest and these alone are sufficient to demand that what can properly be done by this way shall not be done by any other.

215 LAKE AVENUE.

DISCUSSION.

Dr. A. B. MILLER, Syracuse: The paper read by Dr. Jones is one certainly of marked interest, treating upon a subject which is, perhaps, engaging the attention of the surgical profession as much as any other at the present time. It was a question in my mind, how the author was going to reconcile the conditions of vaginal celiotomy. I readily see now that his method is getting into the abdominal cavity by another route and probably strictly is not a celiotomy. This method is the one that is being practised now by abdominal and gynecological surgeons in preference to the old method of removing diseased structures through the abdominal opening, with a much less mortality attending. While, theoretically, the old method would appeal to our reason as affording greater opportunity for recovery, the mortality was large and practical experience teaches us that the pathological pelvic conditions can be successfully removed through the vaginal route and that a much less mortality attends this procedure.

I again wish to congratulate Dr. Jones in bringing this subject before the society and the able manner in which he has treated it.

